# Comprehensive two-dimensional gas chromatography–mass spectrometry combined with multivariate data analysis for pattern recognition in Ecuadorian spirits

**DOI:** 10.1186/s13065-018-0470-x

**Published:** 2018-10-11

**Authors:** Noroska Gabriela Salazar Mogollón, Guilherme Lionello Alexandrino, José Rafael de Almeida, Zulay Niño-Ruiz, José Gregorio Peña-Delgado, Roldán Torres-Gutiérrez, Fabio Augusto

**Affiliations:** 1 Ikiam-Universidad Regional Amazónica, Km 7 Via Muyuna, Tena, Napo Ecuador; 2 Institute of Chemistry, State University of Campinas, Cidade Universitária Zeferino Vaz, Campinas, SP 13083-970 Brazil; 3 Departamento de Investigación, Universidad Estatal de Bolívar (UEB) Campus Universitario Laguacoto II, Km ½, via San Simón, Cantón Guaranda, Provincia Bolívar Ecuador

**Keywords:** Multiway Principal Component Analysis, Spirits beverages, Comprehensive two-dimensional gas chromatography (GC × GC), Solid phase microextraction, Fisher-ratio

## Abstract

The current methodology used in quality control of Ecuadorian beverages such as Pájaro azúl, Puro and Pata de vaca is carried out by using conventional gas chromatography; however, it does not allow the fingerprinting of these Ecuadorian spirit beverages and their possible cases of adulteration. In order to overcome this drawback, comprehensive two-dimensional gas chromatography–mass spectrometry (GC × GC–MS) was combined with multivariate data analysis, revealing that compounds like citronellal, citronellol, geraniol, methyl anthranilate, (−)-trans-α-bergamotene, (−)-cis-α-bergamotene and d-limonene can be considered key elements for pattern recognition of these traditional beverages and product adulteration cases. Thus, the two-dimensional chromatographic fingerprints obtained by GC × GC–MS coupled with chemometric analysis, using Principal Component Analysis and Fisher-ratio can be considered as a potential strategy for adulteration recognition, and it may used as a quality assurance system for Ecuadorian traditional spirits.

## Introduction

Ancestral and typical liquors have always been an important part of the culture in Ecuador. Beverages such as Pájaro Azúl, Puro and Pata de Vaca are prepared nationwide, and the recipes of these artisanal spirits have remained throughout the centuries. These beverages are distilled liqueurs obtained directly from the raw juice of unrefined sugar cane, whose production process begins with the extraction of the cane juice followed by its fermentation during 96 h at 26 °C approximately [1]. Then, the fermented juice goes through a second distillation, which results in the Puro beverage that contains 70% of alcohol approximately. Afterwards, fruits, herbs and/or animal legs may be added to the Puro, and a third distillation is performed to obtain other beverage variants. For example, chicken legs and some specific herbs are added to create Pájaro azul, while beef legs and other fruits and herbs are used for making of Pata de vaca, in agreement with local references available in databases from Ministerio de Industrias y Productividad (MIPRO) and Ministerio de Agricultura, Ganadería, Acuacultura y Pesca (MAGAP). Therefore, the sensorial characteristics of each beverage are unique and strongly dependent on the raw materials used throughout the whole production process [2, 3].

Ecuador produces about 36.500 L of liquor per day, and most of the artisanal production of liquors occur in the province of Bolivar where there are approximately 600 associated producers. From 30 to 40% of these producers also work independently, and almost 900 families obtain their income from these spirits beverage commerce. Currently, the determination of the quality of beverages, as well as the concentration limits for congeners and some toxic compounds are determined by the Ecuadorian norm INEN 2014 [4]. The analyses are performed by conventional gas chromatography, which searches for the presence of some target compounds only such as acetaldehyde, methanol, isopropanol, *n*-propanol, ethyl acetate, iso-butanol, *n*-butanol, isoamyl alcohol, *n*-amylic and furfural [4]. Different from some well-known spirits samples (tequila, whisky, rum, cachaça, among others) a great variety of other compounds that are strongly related to the organoleptic and aromatic properties of the Ecuadorian spirit beverages has never been investigated so far. This investigation can be achieved by extending gas chromatographic analysis to the identification of other volatile organic compounds like esters, terpenes, aldehydes and higher alcohols [1, 3, 5]. In order to avoid counterfeiting (or counterfeits), it is very important to identify the most relevant compounds closely associated to the origin of the spirits beverages in an attempt to discover possible adulterations, because some non-associated producers may perform adulteration of the beverages due to commercial reasons. For instance, authentic Pájaro Azul and Pata de vaca beverages present a blue and pale yellow color respectively, but the addition of colorants or other products with similar colors can alter the resulting beverage. Therefore, an analytical method that is capable of providing a complete characterization of these Ecuadorian spirits, as well as distinguishing among these original varieties from counterfeits has become necessary.

Headspace solid-phase microextraction (HS-SPME) has being recognized as a successful sample preparation procedure for analysis of volatile compounds using gas chromatography, mainly because of its advantages such as experimental simplicity and the absence of solvent, thus having several applications in the quality control in the food industry. To illustrate this, HS-SPME coupled with GC has been successfully used to determine relevant volatile aromas for the quality of cachaça, beers, wines, tequilas and rums [5–10].

However, due to the complex variety of the volatiles presented in these beverages, coelutions are generally observed in their corresponding chromatograms. Consequently, a comprehensive two-dimensional gas chromatography–mass spectrometry (GC × GC–MS) is a powerful tool for overcoming this drawback, providing higher detectability as well as higher chromatographic separation efficiency.

In GC × GC, two capillary columns containing preferably orthogonal separation capabilities are connected through a modulator, which concentrates the eluate coming from the end of the first column (1D) and then reinjects this eluate in a narrower band into the head of the second column (2D) [11–14].

The great advantage of using HS-SPME along with GC × GC–MS is the possibility of an enhanced characterization of the volatile compounds contained in the samples, but the high amount of these extracted compounds that are chromatographed can make the visual discrimination among several samples an extremely difficult task. For example, Cardeal et al. [5], identified the compounds that are formed during the production of cachaça when analyzing several fractions of this distillate using HS-SPME and GC × GC–TOFMS. However, the authors affirmed that the discrimination between the fractions and the identification of their most relevant variables demanded too much time, and that the type of wood or time of fermentation could not be identified.

Chemometric analysis, mainly principal components analysis (PCA) has proved a powerful technique for the extraction of patterns from large multivariate datasets such as chromatographic data, allowing the identification of chemically similar samples as well as the most relevant variables that are responsible for these clusterings. PCA systematically decomposes the data matrix into eigenvectors and eigenvalues that describe the different sources of variation, according to their respective percentages of the total variance occurring in the multivariate data. Thus, when PCA is applied to entire chromatographic datasets, all the peaks that best explain the variability of the samples can be identified and analyzed more comprehensively, contrary to the univariate data analysis approach in which each peak has to be evaluated separately, restricting, thus, data analysis to only some conventional compounds [15]. However, the high complexity of the data coming from the large amount of variables, commonly occurring in chromatographic analysis of biological samples, can make the interpretation of all the compounds responsible for the patterns from PCA a non-trivial task. Therefore, variable selection strategies aiming to reduce the data complexity toward preserving only the most relevant variables that discriminate between the groups of samples are of paramount importance, and the use of the multivariate Fisher-ratio approach may achieve this goal. Herein, successive one-way Anova is performed in each variable of the data while discriminating samples between their corresponding classes, and non-important variables disturbing the discrimination of the samples can be excluded from the data [16–18]. The data dimensionality reduction to only the most relevant variables clarifies the interpretation of the role of each compound in the sample, and it can be obtained by PCA [3, 10]. Orujo samples were characterized according to the geographical origin of the grapes and the distillation system used for the elaboration of the spirits through the GC-HS-SPME profiles and PCA [7]. HS-SPME was combined with GC × GC–TOFMS in order to characterize Bianco and Giallo Moscatel sparkling wines, using Fisher-ratio and multiway PCA, observing the clear difference between the types of wine due to the higher concentration of terpenes and norisoprenoids in the Giallo type [3]. In another similar study, compounds like 2,3-butanediol, 4-carene, 3-penten-2-one, diethyl succinate, β-santalol, diethyl malonate, dihydro-2(3H)-thiophenone, tetrahydro-2(2H)-pyranone, C9 alcohols, 3-methyl-2(5H)-furanone, ethyl 9-decenoate and nerol, were found in such wines such as Cabernet Sauvignon, Merlot, Chardonnay and Sauvignon Blanc as potential markers of grape variety [10].

Taking into consideration all the aforementioned, the main goal of this study was to develop a reliable analytical method based on HS-SPME GC × GC-QMS aiming for the complete characterization of the Ecuadorian spirits beverages Pájaro azul, Pata de vaca and Puro. In order to achieve this goal, chromatographic data was combined with Fisher-ratio to identify the most relevant and distinguishing compounds among these types of beverages, and (multiway) PCA was used to determine straightforwardly the relations among the profiles of these relevant compounds in each type of beverage to discover potential chemical markers in their qualities.

## Materials and methods

### Chemical and materials

#### Spirit samples

Six different samples of the beverage Puro, six of Pata de vaca and six of Pájaro azúl were taken for the analysis. All the samples were obtained from Guaranda, central state of Ecuador, and these spirit beverages were produced following a traditional artisanal methodology.

#### Reagents and materials

A series of C8–C22 *n*-alkanes (Sigma-Aldrich-St. Lois, MO, USA) was used for the determination of the 1D linear temperature programmed retention indices (LTPRI), additionally hexane and heptane were used in order to calculate with high precision minors alkanes. The HS-SPME procedures were performed using a SPME fiber coated with 50/30 µm divinylbenzene/Carboxen on poly(dimethylsiloxano) (DVB/CAR/PDMS) (Sigma-Aldrich). Septum-sealed Pyrex vials of 20.00 mL (Wheaton science Products-Millvine, NJ, USA), volumetric flask of 50.00 mL and magnetic stirrers were also used during the sample preparation procedures (Sigma-Aldrich).

#### HS-SPME sample preparation

An aliquot of 5.00 mL of the spirit samples was diluted with water in a volumetric flask of 50 mL containing 2.5 g of sodium chloride [1, 5]. Then, 10 mL of this solution was transferred into a 20.00 mL septum-sealed Pyrex vials, and the SPME fiber was exposed in the headspace during 20 min, at T = 60 °C and magnetic stirring (600 rpm). For the retention indices determination, samples were spiked with 5 µL of a C8–C22 *n*-alkanes standard mixture [19]. The extracted compounds were immediately desorbed into the GC injector at 250 °C for 3 min.

#### Equipment

The analyses were performed on a lab-made GC × GC-QMS prototype based on a QP2010 + GC (Shimadzu Corp, Tokyo, Japan) fitted with a split/splitless injector and equipped with a miniaturized sealed two-stage cryogenic modulator that provided cold (T = − 196 °C) and hot (T = 250 °C) jets that were controlled by solenoid valves (ASCO, Florham Park, NJ—USA) and a 8-bit Duemilanove microcontroller board (Arduino, Ivrea, Italy) [20]. The modulation period was set to 6.0 s. The column set consisted of a 25 m × 0.25 mm × 0.25 µm HP-5 MS (Agilent Technologies—Palo Alto, CA, USA) column (1D) fitted with a 1 m × 0.10 mm × 0.10 µm SupelcoWax 10 column (Sigma-Aldrich), as the second dimension (2D). The oven temperature programming was initially set to T = 35 °C (t = 5 min), then it was raised to 210 °C at 3 °C/min, next to T = 240 °C at 40 °C/min and finally holding for 10 min. The injection port and transfer line were kept at T = 250 °C, using hydrogen as carrier gas at initial flow of 0.6 mL/min. The MS ionization source was set to 200 °C and the mass scan range was set from m/z 40 to 487 Da, at acquisition rate of 20 Hz. The peaks identification was performed using the NIST 2010 (NIST, Gaithersburd—MD, USA) and the FFNSC (Chromaleont, Messina, Italy) spectra libraries combined with the LTPRI inspections. All the analyses were performed in duplicate. The raw two-dimensional chromatograms were generated using the GCImage software (Zoex Corp., Houston, TX, USA).

#### Multivariate analysis

The raw unfolded GC × GC–Q(TIC)MS chromatograms were firstly converted to .txt files and then imported into Matlab^®^ R2013b software (MathWorks, Natick-MA, USA). Next, the chromatographic peaks were aligned using the *icoshift* algorithm [21] and the Fisher-ratio was performed throughout the aligned chromatograms using an in-house routine written in Matlab. The PCA was performed in the mean-centered unfolded chromatograms containing only the selected peaks obtained previously from the Fisher-ratio results, using the software Pls_Toolbox v. 8.1.1. for Matlab (Eigenvector Research Inc., Wenatchee—WA, USA). The chromatographic loadings extracted from PCA were re-folded to the original two-dimensional chromatographic structure for visualization and interpretation.

## Results and discussion

**Fig. 1:**
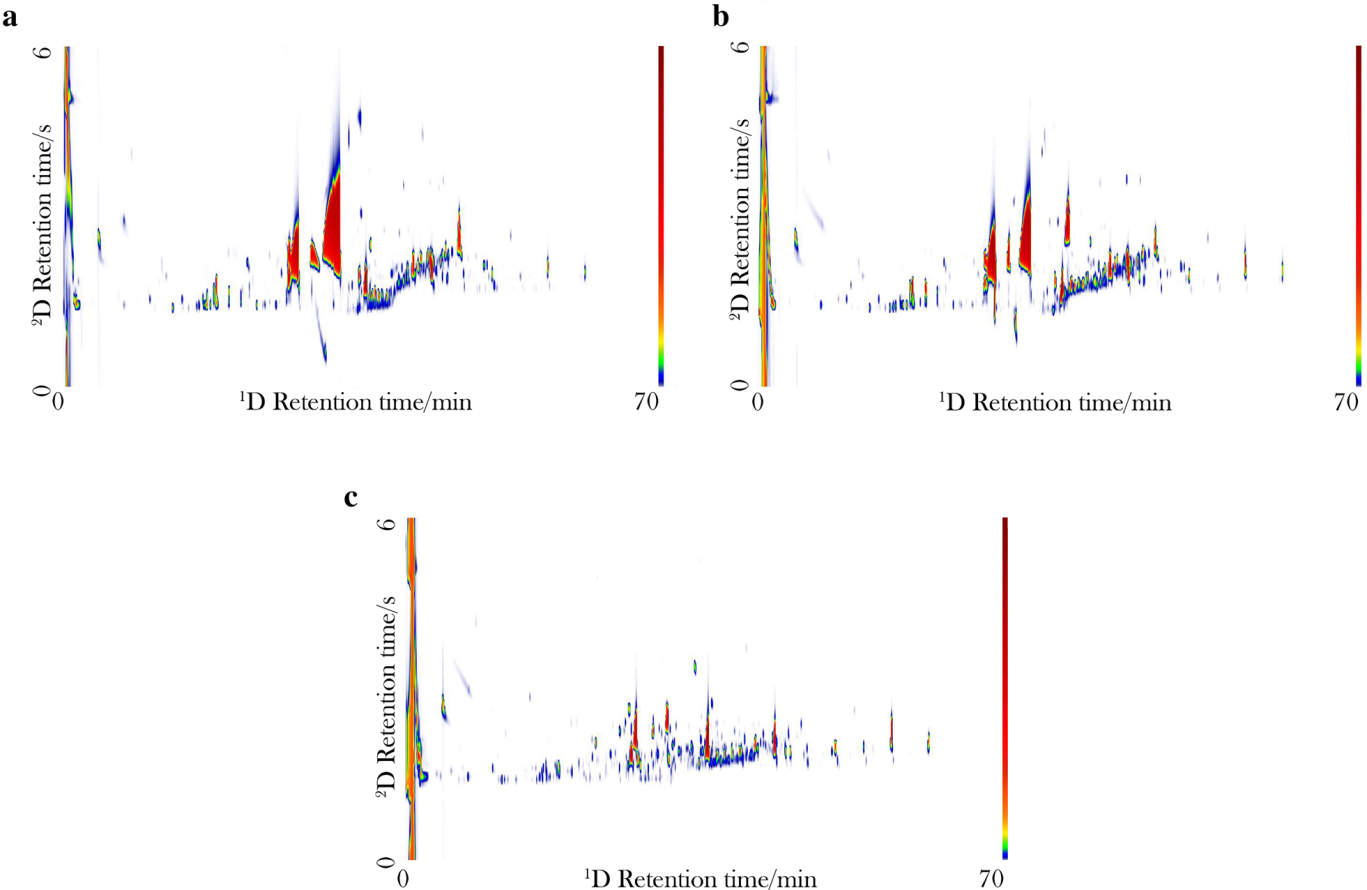
GC × GC-QMS colour plot obtained from the Ecuadorian spirits beverages using HS-SPME–GC × GC-QMS: **a** Pata de vaca, **b** Pájaro azul, **c** Puro

The conditions for the extraction of the compounds in the spirits were adapted from a previous research [5], in which the 6.0 s modulation period was suitable for the proper chromatographic separation in 2D without jeopardizing the efficiency in 1D, during the analysis around 150 approximately were detected. However, 100 compounds were identified which are the responsible for the differentiation of the samples. Figure 1 shows the aligned unfolded chromatograms of the samples, whose variance may be mostly attributed to the chemical diversity in the beverages that is the result of the different ingredients used during the preparation of each type of beverage.

Additionally, the profiles of the chromatographed compounds are a consequence of the physicochemical properties of the DVB/CAR/PDMS fiber that selectively extract polar and non-polar compounds. A clear difference between the samples of Puro and Pájaro azul, and between Puro and Pata de vaca can be easily noticed in Fig. 1, while distinguishing Pájaro azul from Pata de vaca is much more difficult due to the highly similar fingerprints between the samples of these beverages. Therefore, the use of multivariate data analysis for pattern recognition was required to achieve this goal, obtaining a better discrimination amongst the types of beverages by firstly selecting only the most relevant compounds for the discrimination, and afterwards computing the Fisher-ratio for each peak, followed by multiway PCA. The compounds were tentatively identified according to the MS library matching and retention index criteria, in which the uncertainty threshold of 3% was considered reasonable (Table 1).

**Table 1:** Compounds identified in the Ecuadorian spirits beverages using GC × GC-QMS

#	Compounds	LTPRI Exp	LTPRI Lit	Pájaro azul	Pata de vaca	Puro	CIS (*)
1	Ethanol	460	463	x	x	x	
2	2-Propanol	480	482	x	x	x	
3	2-Methyl-1-propanol	630	628	x	x	x	
4	2-Butanol	579	581	x	x	x	
5	Ethyl acetate	644	647	x	x	x	
6	*n*-Butanal	643	650	x	x	x	
7	*n*-Butanol	660	662	x	x	x	
8	Ethyl propanoate	708	686	x	x	x	
9	2-Methyl-1-butanol	697	697	x	x	x	
11	3-Methyl-1-butanol	754	734	x	x	x	
12	3-Hydroxybutanal	768	770	x	x	x	
14	*n*-Pentanol	760	766	x	x	x	
15	2-Methyl-1-butanol	729	731	x	x	x	
16	Hexanal	799	801	x	x	x	
17	Ethyl 2-hydroxypropanoate	811	814	x	x	x	
18	Furfural	831	845	x	x	x	
19	*n*-Hexanol	861	860	x	x	x	
20	Isopentyl acetate	872	871	x	x	x	
21	Ethyl pentanoate	884	887	x	x	x	
22	Heptanal	902	906	x	x	x	
23	2-Heptanol	910	913	x	x	x	
24	1-Heptanol	980	981	x	x	x	
25	1*S*-α-Pinene	945	948	x	x		
26	2-Hydroxy-3-pentanone	974	960	x	x	x	
27	2(R)-Octanol	973	976	x	x	x	
28	β-Pinene	980	978	x	x		
29	Ethyl hexanoate	985	984	x	x	x	
30	*n*-Octanal	1002	1005	x	x	x	
31	Carene	1008	1009	x	x		
32	α-Terpinene	1018	1017	x	x		
33	*p*-Cymene	1021	1025	x	x		
34	1,3,8-*p*-Menthatriene	1023	1029	x	x		
35	d-Limonene	1027	1030	x	x		
36	β-Ocimene	1046	1046	x	x		
37	γ-Terpinene	1056	1058	x	x		
38	2-Cyclopenten-1-one	1058	1060	x	x	x	
39	*n*-Octanol	1073	1076	x	x	x	
40	2-Nonanol	1079	1078	x	x	x	
41	Linalool	1088	1081	x	x		
42	Ethyl heptanoate	1084	1083	x	x		
43	Terpinolene	1085	1086	x	x		
44	2-Nonanone	1090	1093	x	x	x	
45	*n*-Nonanal	1106	1104	x	x	x	
46	2,4-Dimethylanisole	1112	1110	x	x		
47	Acetophenone	1100	1142	x	x		
48	*p*-Menthane	1217	1148	x	x		
49	*p*-Cumenol	1112	1149	x	x		
50	1-Nonanol	1160	1159	x	x	x	
51	Citronellal	1161	1161	x	x		
52	Ethyl benzoate	1170	1170	x	x		
53	Estragole	1170	1172	x	x		
54	Terpinen-4-ol	1182	1180	x	x		
55	Diethyl succinate	1180	1183	x	x	x	
56	Methyl salicylate	1192	1192	x	x		
57	Ethyl octanoate	1200	1202	x	x		
58	*p*-Propyl anisole	1205	1207	x	x		
59	Citronellol	1225	1228	x	x		
60	(Z)-Anethole	1255	1253	x	x		*
61	Geraniol	1257	1255	x	x		
62	Phenethyl acetate	1260	1257	x	x		
63	1-Decanol	1263	1258	x	x	x	
64	Ethyl-non-3-enoate	1270	1272	x	x	x	
65	(E)-Anethole	1289	1288	x	x		*
66	Undecen-2-ol	1294	1295	x	x	x	
67	Undecan-2-one	1297	1296	x	x	x	
68	Propyl octanoate	1302	1300	x	x	x	
69	2-Undecanol	1305	1303	x	x	x	
70	4-Propylguaiacol	1320	1313	x	x		
71	Sec-butyl octanoate	1327	1317	x	x	x	
72	4-Allylphenyl acetate	1373	1362	x	x	x	
73	β-Damascenone	1378	1379	x	x		
74	*p*-acetonylanisole	1387	1384	x	x		
75	Ethyl-dec-9-enoate	1390	1389	x	x	x	
76	Ethyl decanoate	1396	1399	x	x		
77	*n*-Dodecanal	1409	1402	x	x	x	
78	Methyl anthranilate	1410	1410	x	x		
79	(−)-cis-α-bergamotene	1417	1416	x	x		*
80	β-Caryophyllene	1423	1424	x	x		
81	3-Methylbutyl octanoate	1448	1446	x	x	x	
82	Isopentyl octanoate	1445	1449	x	x		
83	β-Farnesene	1460	1452	x	x		*
84	(−)-trans-α-bergamotene	1456	1458		x		*
85	α-Farnesene	1458	1460	x	x		*
86	*n*-Dodecanol	1475	1473	x	x	x	
87	Ethyl undecanoate	1495	1498	x	x		
88	β-Bisabolene	1510	1508	x	x		
89	Nerolidol	1563	1564	x	x		
90	*n*-Tridecanol	1578	1575	x	x	x	
91	Ethyl dodecanoate	1595	1598	x	x		
92	iso-Amyl *n*-decanoate	1620	1615	x	x		
93	*n*-Tetradecanol	1679	1677	x	x	x	
94	Foeniculin	1681	1679	x	x		
95	α-Bisabolol	1683	1688	x	x		
96	Ethyl tetradecanoate	1775	1794	x	x	x	
97	Ethyl pentadecanoate	1858	1878	x	x	x	
98	Ethyl hexadecanoate	1940	1978	x	x	x	
99	Ethyl heptadecanoate	2050	2077	x	x	x	
100	Ethyl octadecanoate	2050	2177	x	x	x	

CIS (*): Compounds identified by structuration

**Fig. 2:**
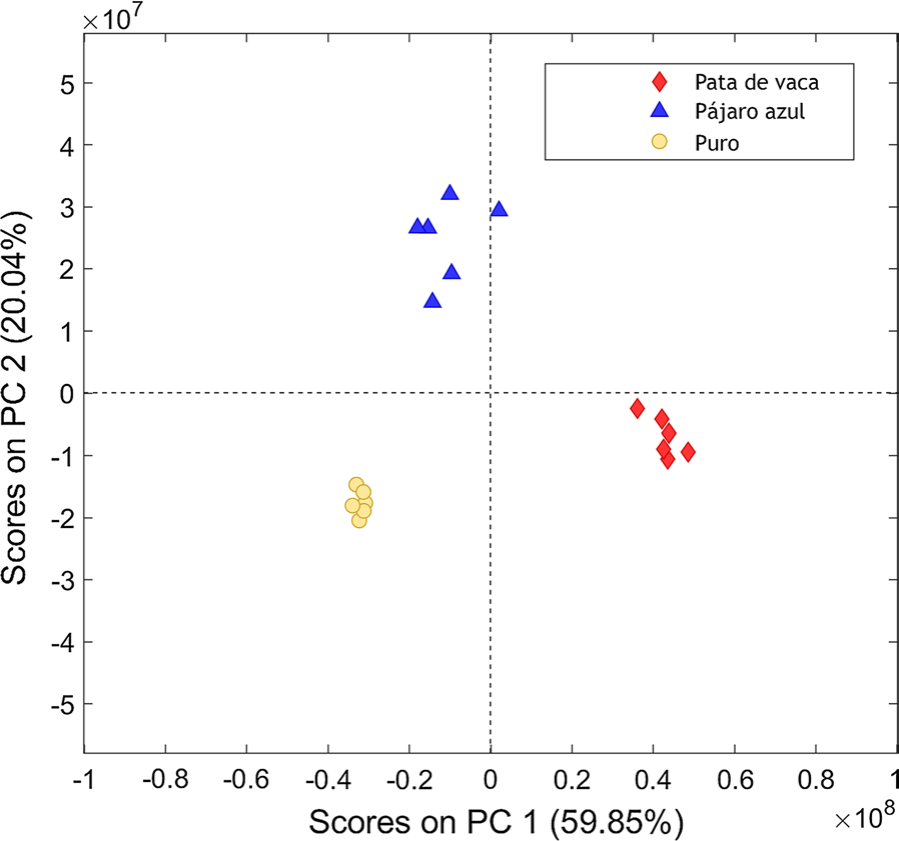
PC1 vs PC2 scores plot obtained from the Ecuadorian spirits beverages

The two-dimensional structure of the chromatograms was also used to support the identification of homologous compounds. Moreover, the multiway PCA provided two factors that explained 59.85% (PC1) and 20.04% (PC2) of variance in the data, and no outliers were detected. The three different types of beverages could be distinguished in the reduced subspace defined by the PCs (Fig. 2).

**Fig. 3:**
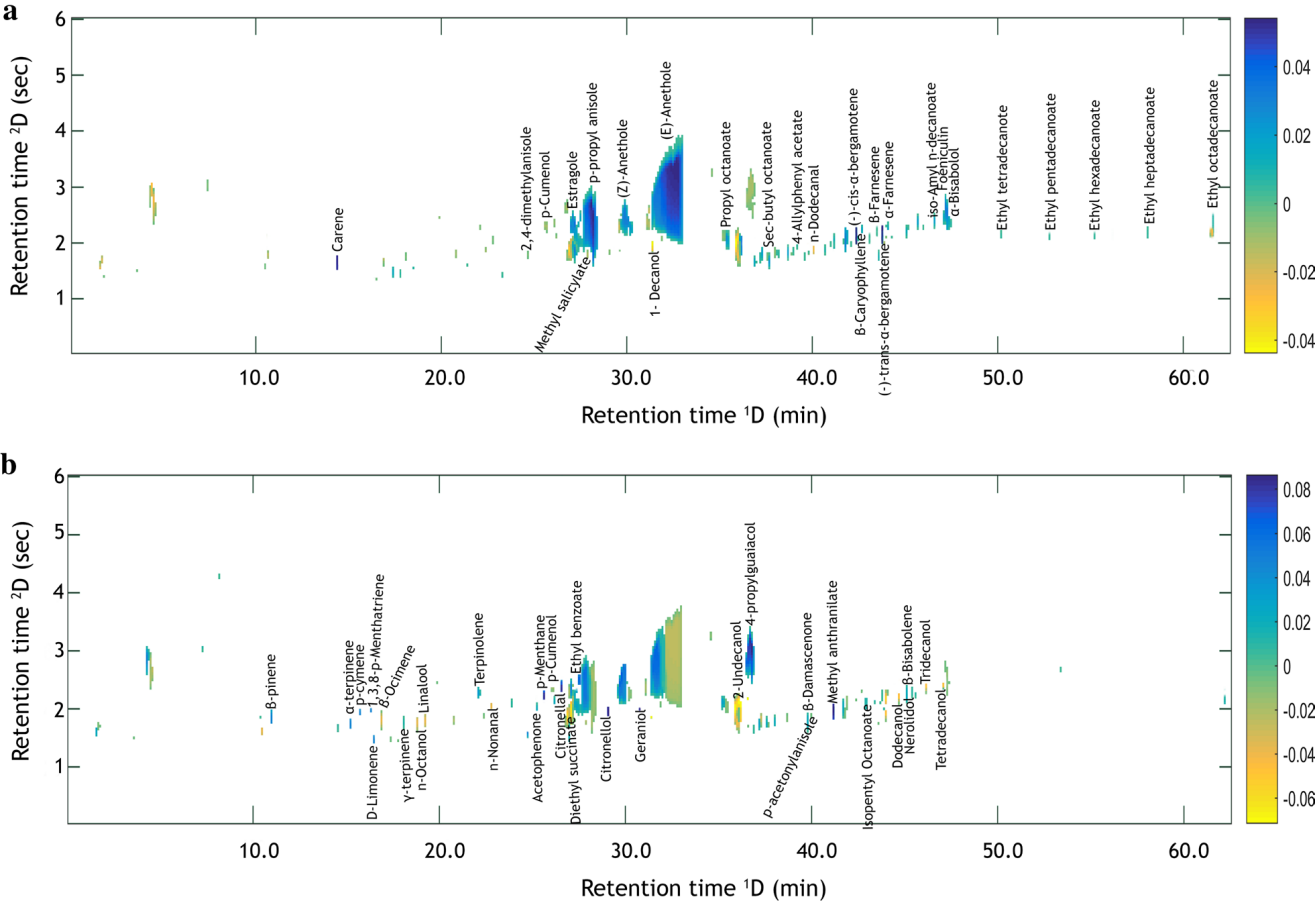
**a** Two-dimensional chromatographic loadings from PC1 and **b** PC2

The most important compounds related to the discrimination among the samples for each factor were identified in the loadings plots of the model depicted in the Fig. 3. On the one hand, the compounds found in the negative loadings were responsible for the differentiation between the samples, and these were identified in the plot with shades of yellow with a scale from 0 to − 0.04. On the other hand, the compounds in the positive loadings were common among the samples and identified in the plot with shades of blue with a scale from 0 to 0.08.

In general, the compounds identified in all the samples belong to the family of alcohols, aldehydes, ester and acetates. Essentially, compounds 2-methyl-1-propanol, 2-propanol, ethyl acetate, *n*-Butanal, 3-methyl-1-butanol, 2-methyl-1-butanol, hexanal, 3-hydroxybutanal, furfural, isopenty acetate and heptanal were tentatively identified in the positive loadings from the PC1, which are the most volatile compounds recognized by their negative organoleptic contributions and described as “spicy” or “solvent-like” and produce toxic effects [1, 8]. Short chain alcohols such as *n*-butanol, *n*-pentanol, *n*-hexanol, *n*-heptanol and 2-heptanol (which are normally associated with green flavor) were also identified, which may result in prejudicial sensorial characteristics for the beverage when found in higher concentrations [5, 22–24]. However, these compounds were identified in higher concentrations in both Pájaro azul and in Pata de vaca than in Puro, according to the loadings plots (Fig. 3a).

Esters compounds such as ethyl heptanoate, ethyl octanoate, ethyl nonanoate, ethyl decanoate, ethyl undecanoate, ethyl dodecanoate, ethyl tetradecanoate, ethyl pentadecanoate, ethyl hexadecanoate, ethyl heptadecanoate and ethyl octadecanoate were identified, which are present in many alcoholic beverages such as tequila, rum and cachaça [7, 23, 25]. These compounds are usually associated with fruity and pleasant attributes, and they were found in higher concentration in both Pata de vaca and in Pájaro azul than in Puro. The preparation process of Pata de vaca and Pájaro azul beverages supports this result, as chicken legs are commonly used in Pájaro azul, while beef legs are added to Pata de vaca. Therefore, a higher concentration of these compounds can be the result of esterification reactions between some animal saturated fatty acids and the ethanol contained in the beverage, as well as the heating occurring during the distillation and production processes.

The positive loadings in PC1 also show the compounds 2,4-dimethylanisole, estragole, foeniculin, *p*-propyl anisole, (Z)-anethole and (E)-anethole in higher concentration in Pata de vaca than in Pájaro azul. Despite the fact that both beverages contain the same quantity of anise, Pájaro azul contains a greater number of additional components like fruits, which may result in a dilution of the compounds responsible for the anise flavor. In addition, it is worth considering that the alcohol content in Pata de vaca is 45% approximately, while the alcohol content in Pájaro azul is 40%, and that both beverages are the result of the same distillation cut. In addition, some plants used exclusively during the preparation of Pata de vaca can contribute to their constituents, increasing the concentration of these compounds in this beverage. To illustrate this, compounds such as *p*-cumenol, methyl salicylate, 4-allylphenyl acetate, propyl octanoate, β-caryophyllene, α-farnesene, β-farnesene, iso-amyl *n*-decanoate, sec-butyl octanoate and α-bisabolol were also present in Pájaro azul and Pata de vaca (higher concentrations), and these compounds are generally found in plant extracts, which are mainly used in Pata de vaca according to its artesian recipe.

On the other hand, the compounds carene, (−)-trans-α-bergamotene and (−)-cis-α-bergamotene, which are characteristic of some herbs and plants that may be used during the beverage production, were identified only in the positive loadings of Pata de vaca. Carene is particularly characteristic of rosemary that is a herb used in the preparation of Pata de vaca, as well as carrots, which are associated with the (−)-trans-α-bergamotene and (−)-cis-α-bergamotene compounds responsible for the yellow color of this beverage, and thus they can be considered origin markers [26, 27] (Fig. 3a).

In the negative chromatographic loadings in PC1, compounds such as 1-decanol, and *n*-dodecanal were found in higher concentration in Puro and Pájaro azul, and they are associated with toxic effects [7, 23].

The positive loadings in PC2 refer to the compounds contained in Pata de vaca and Pájaro azul and were found in higher concentrations; these were β-pinene, linalool, α-terpinene, *p*-cymene, 1,3,8-*p*-menthatriene, β-ocimene, γ-terpinene, terpinolene, acetophenone, ethyl benzoate, *p*-menthane, 4-propylguaiacol, β-damascenone, isopentyl octanoate, β-bisabolene, nerolidol and p-acetonylanisole (Fig. 3b). These compounds have a very high significance in the positive organoleptic characteristics associated with pleasant aromas of fruits, and they also play an important role in the flavour of beverages as wine [10], these compounds were found mainly in Pájaro Azul whose production requires a great amount of fruits.

Additionally, the identification of the compounds d-limonene, methyl anthranilate, citronellal, citronellol and geraniol in Pájaro azul in high concentration may indicate markers of origin (Fig. 3b). The typical blue color of this beverage corresponds to the ancestral recipe in which the artisans add leaves of tangerine to provide this color. While d-limonene and methyl anthranilate are characteristic compounds in citrus fruits such as tangerines, oranges and lemons, citronellal, citronellol and geraniol are compounds found mainly in Citronella grass, which is a herb used during its production. The presence of these compounds help to perform the quality control of authentic Pájaro azul, as opposed to counterfeits in which the blue color is due to some colorants in the beverage in order to avoid expenses and raw material consumption, colorants in the beverage that are not reported in sugar cane [28–30]. Furthermore, the higher molecular weight of the compounds *n*-octanol, *n*-nonanal, 2-undecanol, *n*-dodecanol, *n*-tridecanol and *n*-tetradecanol identified in high concentration in the negative PC2 loadings belong to the Puro as well as, and these compounds are related to the poor aroma quality of this beverage. Diethyl succinate was also identified, which is a secondary compound resulting from fermentation and provides some pleasant flavor [23].

Performing quality control during the distillation process with the purpose of eliminating toxic compounds, but maintaining the compounds associated with the flavors at the same time, proves an interesting approach from the commercial perspective. Specifically, techniques using high chromatographic resolution such as GC × GC, along with (multiway) PCA for the identification and characterization of volatile profiles of these ancestral selected spirits provided a suitable and time-efficient tool in order to assure quality control during their production. These techniques also ensured the presence of their most important constituents, especially those that have a great influence on the chemical and physical characteristics of these beverages. Finally, this suggests that the monitoring of these compounds should be part of the routine protocols to ensure quality and to avoid the addition of any other components in the recipe that may affect the characteristics of the final product.

## Conclusions

Comprehensive two-dimensional gas chromatography along with MPCA allowed the discrimination between three Ecuadorians artisan spirits, characterizing the volatile profiles of each them, in order to measure their qualities. MPCA along with Fisher ratio allowed to perform a tentative identification of the most important compounds for the discrimination of the beverages, as well as the detection of the compounds that can considered marker of origin. The monitoring of these compounds may avoid counterfeiting practices, mainly those related to the substitution of the original products that contain the essential components responsible for their organoleptic properties, according to the ancestral recipe. In this study, Pájaro azul and Pata de vaca were found to be significantly different from Puro, but they were very similar to each other to the extent of becoming almost impossible to truly distinguish each other only by simple visual inspection. However, the target analysis of the main compounds such as citronellal, citronellol, geraniol, methyl anthranilate, carene, (−)-trans-α-bergamotene, (−)-cis-α-bergamotene and d-limonene can provide the basic chemical differences between these spirits, since they have low concentrations in these beverages. GC × GC–MS became an alternative to the proper separation and detection of such compounds; As a result, the two-dimensional chromatographic fingerprints obtained by GC × GC–MS coupled with chemometric analysis using MPCA and Fisher Ratio proved valuable tools for the characterization and quality inspection of these spirit beverages.
